# Ivabradine and Atrial Fibrillation Incidence: A Nested Matching Study

**DOI:** 10.1016/j.jacadv.2025.102282

**Published:** 2025-10-28

**Authors:** Kibum Kim, Jasmeen Keur, Halie Anderson, Przemysław B. Radwański, Mark A. Munger

**Affiliations:** a Department of Pharmacy Systems and Outcomes and Policy, University of Illinois at Chicago, Chicago, Illinois, USA; b Department of Pharmacotherapy, University of Utah Health, Salt Lake City, Utah, USA; c Department of Physiology and Cell Biology, The Ohio State University, Columbus, Ohio, USA; d The Frick Center for Heart Failure and Arrhythmia, Dorothy M. Davis Heart and Lung Research Institute, College of Medicine, The Ohio State University Wexner Medical Center, Columbus, Ohio, USA; e Department of Internal Medicine, University of Utah Health, Salt Lake City, Utah, USA

**Keywords:** atrial fibrillation, health care, heart failure, ivabradine, outcomes assessment

## Abstract

**Background:**

Ivabradine (IVB) has been utilized for managing hospitalization risk among patients with heart failure (HF). The risk of atrial fibrillation (AF) among IVB patients is poorly understood.

**Objectives:**

The purpose of this study was to assess the risk of developing AF in HF patients receiving IVB.

**Methods:**

A retrospective observational comparative study was performed using a health plan claims database. A new AF diagnosis was compared between adult HF patients receiving IVB vs no-IVB controls (CTRs) over 180-day follow-up period. Eligible IVB subjects were free from AF diagnosis before IVB index date, longer than 6 months. Incidence-density sampling was performed to select matched CTRs based on clinical characteristics and time from the initial HF diagnosis. Cox proportional hazards regression model was used to determine AF risk in patients who did or did not receive IVB.

**Results:**

Of the 153 IVB and 4,494,305 CTRs meeting the AF-naive HF status, the analytic cohort of 107 IVB and 321 matched controls were created. The groups were well matched for age: 52.9 ± 11.3 (IVB) vs 53.5 ± 11.8 years, sex: 57% male, heart failure with reduced ejection fraction diagnosis: 66.4%, comorbidities and goal-directed medical therapy: ß-adrenergic blockers 89.7%, angiotensin-converting enzyme inhibitors/angiotensin receptor antagonists/sacubitril/valsartan 79.4%, and mineral corticoid antagonists 39.3%. The adjusted HR (95% CI) for developing AF in those receiving IVB was 7.293 (4.985-10.668).

**Conclusions:**

The risk of AF over 180-day period was higher among HF patients who received IVB vs those who did not receive IVB. Further studies are warranted to understand this association, including the impact of unmeasured risk factors and confounding by indication.

Atrial fibrillation (AF) is the most common cardiac arrhythmia, with its prevalence steadily increasing worldwide. This rise can be attributed to the growing burden of cardiovascular disease, the increasing prevalence of metabolic disorder risk factors, and by drug-induced mechanisms.^1^^,^^2^ AF substantially contributes to morbidity, mortality, and medical expenditures.^3^ Health care total costs including out-of-pocket, medical utilization, prescription drugs, and incremental costs are all substantially higher with AF vs non-AF patients.^4^ AF is preventable, early detection and appropriate treatment can substantially reduce AF-adverse outcomes.

Ivabradine (IVB) is a hyperpolarization-activated cyclic nucleotide-gated (HCN) channel blocker.^5^ The agent through inhibition of the cardiac pacemaker current (HCN-mediated funny current) [I_f_] current, causes spontaneous depolarization in the sinoatrial node that regulates heart rate.^6^ IVB is indicated for the treatment of NYHA functional classification II-IV chronic heart failure (HF) patients in sinus rhythm, in whom heart rate is ≥75 beats/min in combination with goal-directed medical therapy, including beta-adrenergic blockade or when beta-adrenergic blockade is contraindicated or not tolerated.^5^ IVB has been associated with an increased incidence of AF through underlying gene mutations in HCN or pathophysiology AF triggers.^7^ In addition, HF measures and IVB pharmacology may modulate the effects of IVB to induce AF.^8^, ^9^, ^10^, ^11^ In a recent meta-analysis of 13 randomized clinical trials with 37,533 patients, AF incidence was significantly higher with IVB vs the control group, all with HF (OR: 1.23; 95% CI: 1.08-1.41). Subgroup analysis of left ventricular ejection fraction vs left ventricular ejection fraction <40% subgroups and small cumulative doses of IVB <100 mg within vs large cumulative dose >300 mg for 1 to 27.8 months were associated with higher AF IVB rates.^12^ In a separate meta-analysis with trial sequential analysis of 40,037 patients, it showed a 15% RR increase in AF with IVB treatment.^13^ However, Holter substudies from the BEAUTIFUL (Randomized Trial of Ivabradine in Patients with Stable Coronary Artery Disease and Left Ventricular Systolic Dysfunction)^14^ and SHIFT (Ivabradine and outcomes in chronic heart failure (SHIFT): a randomised placebo-controlled study)^15^ randomized clinical trials did not report a statistically significant increase in AF episodes, when comparing IVB to control patients at baseline, 1 month, or 8 months.

While studies indicated the increase in the risk of AF after IVB, paucity of the data on the real-world evidence have the conclusion remained unclear, and a large real-world study is warranted. To this end, this real-world retrospective, nested-matched study was undertaken to assess the incidence of AF episodes with IVB and compared the risk with non-IVB controls.

## Methods

### Data and cohort selection

A retrospective nested-matched study was performed using administrative claims from MarketScan Commercial Claims and Medicare Supplemental databases (Merative L.P.). The database consists of both medical and pharmacy claims for individuals covered by employment-based health plans. Each service encounter and procedure record are adjudicated by diagnosis codes, while the database did not contain the mortality data, point of care measures, or laboratory test result information. We utilized data from January 2009 through December 2021. Any information that can assist in identifying patients was removed by the data vendor prior to the investigators’ access to the data, making it impossible to link the data back to a specific individual. Using the databases was deemed exempt from the human subject research review by the University of Illinois Chicago Institutional Review Board.

The patient population was defined by individuals with diagnosis of HF, which has been broadly considered as an indication of IVB and was defined by the presence of the International Classification of Diseases-9th or 10th Revision-Clinical Modification (ICD-9-CM 428.x or ICD-10-CM I50.x, and all subcodes) at any diagnosis position of the encounter. The date of the first HF diagnosis, following a 180-day HF-free baseline period during which patients were continuously enrolled without any coverage gaps, was designed as the “first HF date.” This date marked the start of patient follow-up to define exposure.

The first ever IVB dispensing was labeled as the index exposure. To become eligible, patients had to be continuously enrolled in the database from the First HF. Eligible individuals were free from IVB exposure at any time prior to the index exposure date, including the HF baseline period. If patients had a record of cardiac dysrhythmias defined by ICD-9-CM 427.x or ICD-10-CM I48.x before index exposure or during the HF baseline period, we excluded them from the research cohort. Only adult (≥18 years to no upper age limit) patients were selected as eligible IVB patients.

We used an incidence density sampling approach to select controls who were free from the exposure IVB while they were naive to AF. Of the patients with HF, those who had not been exposed to IVB nor had onset of AF until the corresponding IVB patient received IVB were selected as potential controls. The time from the first HF to the IVB date of the IVB patients were added to the control patients’ first HF date, which was flagged as “assigned index date” for the controls. In theory, patients who received IVB later in the follow-up period could have a chance to be selected as a control prior to their index exposure. For such cases, patient follow-up ended with exposure to IVB.

## Matching and outcome assessment

The IVB–control matching was performed based on the patient characteristics collected over the 180-day period on or before either index exposure or assigned control date. Demographics and patient characteristics assessed during the baseline period included grouped age (<45, 45-64, ≥65 years), sex, year of the index exposure date or corresponding assigned index date, acute coronary syndrome or acute myocardial infarction, hypertension, hyperlipidemia, diabetes, record of hospital admission with HF diagnosis at any diagnosis position of the admission summary, and presence of heart failure with reduced ejection fraction (HFrEF). To reduce the concern on the unobserved or missing diagnosis codes that potentially became a confounders in the IVB-AF association, we also matched the baseline medication classes that indicates the severity or stages of HF progression, including angiotensin-converting enzyme inhibitors, angiotensin receptor blockers, and sacubitril/valsartan under the category of renin-angiotensin inhibitors, adrenergic beta-blockers, mineralocorticoid receptor antagonists, and sodium glucose co-transporter-2 inhibitors. We performed a one-by-three matching with replacement, and each of the listed patient-level characteristics was exactly matched between the IVB and controls.

We assessed the onset of AF as the outcome of this study over the 180-day follow-up period from the index exposure or assigned index date. The outcome of this study was the onset of AF defined by the diagnosis code (ICD-9-CM 427.31, ICD-10-CM I48.0 or I48.1) at any position that adjudicate the medical care encounter. For the time-to-event analysis, the patient follow-up ended with the onset of AF, end of continuous enrollment with any gap, or end of the 180-day follow-up period.

### Statistical analysis

Patients’ characteristics, after incidence density sampling but before matching and after the completion of 1-by-3 matching, were compared between the IVB and controls using descriptive statistics followed by bivariate analysis. The summary statistics for age in years were mean and SD, which were compared using Student’s *t*-test. All other categorical variables were summarized using frequency and percentage. We used chi-square test of independence to test the statistical difference in the patient characteristics between the IVB and controls.

Time to AF onset from the index date was summarized and presented using cumulative incidence from Kaplan-Meier product limit estimates. While matched patients were followed until the onset of AF up to 180 days from the index date, following censoring criteria were applied: end of 180-day follow-up period, end of study data (December 2021), any enrollment gap, and receiving IVB if a patient was enrolled in the cohort as a control. The comparative outcome assessment was performed using a Cox proportional hazards regression model from which HR and 95% CIs were calculated.

## Results

**Figure 1:**
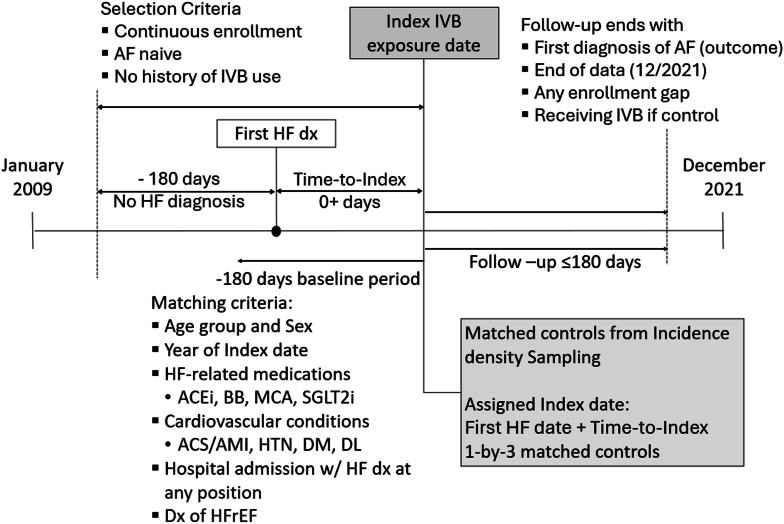
**Cohort Selection and Matching** Index date was defined by the first ivabradine dispensing date. Controls were selected based on time in non-ivabradine since the first AF diagnosis. One-by-three matching was performed based on the patient clinical characteristics and HF-related medication use. Patients were followed up for the analysis of the onset of AF up to 180 days. ACEi = angiotensin-converting enzyme inhibitors; ACS = acute coronary syndrome; AF = atrial fibrillation; AMI = acute myocardial infarction; BB = beta-blockers; DM = diabetes mellitus; DL = dyslipidemia; HF = heart failure; HFrEF = heart failure with reserved ejection fraction; HTN = hypertension; IVB = ivabradine; MCA = Mineralocorticoid antagonists; SGLT2i = sodium-glucose transport protein inhibitors.

Of all the patients with the diagnosis of HF from the database, 153 IVB patients and 4,494,305 controls from incidence density sampling met the continuous enrollment and AF-naive criteria before either IVB or assigned index date (Figure 1). Before matching patient characteristics, IVB patients were younger than the controls with respective mean (SD) year ages of 52.5 (12.2) and 61.2 (16.7). While middle-aged (45-64 years) accounted for 66.7% of the IVB patients, the corresponding proportion among the controls was 49.1%. IVB patients had elevated risks of cardiovascular complications compared to the controls with the respective proportion of acute coronary syndrome/acute myocardial infarction history, hyperlipidemia, HFrEF, and admission with HF diagnosis of 19.6% (vs 8.9%), 60.8% (vs 56.9%), 67.3% (vs 24%), and 51.0% (vs 29.2%). The elevated risk of cardiovascular complications was also shown in the more use of cardiovascular medications with the respective proportions of renin-angiotensin inhibitors, adrenergic beta-blockers, mineral corticoid antagonists, and sodium glucose SGLT2i use of 76.5% (vs 49.7%), 91.5% (vs 44.6%), 46.4% (vs 8.4%), and 8.5% (vs 2.5%). Summary statistics and statistical significance test results are presented in Table 1.

**Table 1:** Baseline Characteristics

Population Characteristics	Before Match			Matched Cohort		
	n = 153	n = 4,494,305	*P* Value	n = 107	n = 321	*P* Value
Age, y, mean (SD)	52.5 (12.2)	61.2 (16.7)	<0.01	52.9 (11.3)	53.5 (11.8)	0.68
Age group, y						
≥65	16 (10.5)	1,683,322 (37.5)	<0.01	7 (6.5)	21 (6.5)	1.00
45-64	102 (66.7)	2,205,633 (49.1)		82 (76.6)	246 (76.6)	
<45	35 (22.9)	605,350 (13.5)		18 (16.8)	54 (16.8)	
Sex			0.19			1.00
Female	71 (46.4)	2,324,114 (51.7)		46 (43.0)	138 (43.0)	
Male	82 (53.6)	2,170,191 (48.3)		61 (57.0)	183 (57.0)	
Index year			0.36			1.00
2015	11 (7.2)	453,472 (10.1)		7 (6.5)	21 (6.5)	
2016	26 (17.0)	1,010,178 (22.5)		22 (20.6)	66 (20.6)	
2017	28 (18.3)	784,983 (17.5)		16 (15.0)	48 (15.0)	
2018	26 (17.0)	740,752 (16.5)		18 (16.8)	54 (16.8)	
2019	23 (15.0)	492,242 (11.0)		16 (15.0)	48 (15.0)	
2020	26 (17.0)	639,391 (14.2)		18 (16.8)	54 (16.8)	
2021	13 (8.5)	373,287 (8.3)		10 (9.3)	30 (9.3)	
ACEIs/ARBs/sacubitril-valsartan	117 (76.5)	2,232,899 (49.7)	<0.01	85 (79.4)	255 (79.4)	1.00
Beta-adrenergic blockers	140 (91.5)	2,006,201 (44.6)	<0.01	96 (89.7)	288 (89.7)	1.00
Mineral corticoid antagonists	71 (46.4)	379,240 (8.4)	<0.01	42 (39.3)	126 (39.3)	1.00
SGLT2i	13 (8.5)	112,699 (2.5)	<0.01	2 (1.9)	6 (1.9)	1.00
Acute coronary syndrome/acute myocardial infarction	30 (19.6)	397,810 (8.9)	<0.01	16 (15.0)	48 (15.0)	1.00
Diabetes mellitus	61 (39.9)	1,370,648 (30.5)	0.01	41 (38.3)	123 (38.3)	1.00
Hyperlipidemia	93 (60.8)	2,559,128 (56.9)	0.41	65 (60.7)	195 (60.7)	1.00
Hypertension	114 (74.5)	3,417,696 (76.0)	0.50	87 (81.3)	261 (81.3)	1.00
Admission with heart failure	78 (51.0)	1,311,290 (29.2)	<0.0001	54 (50.5)	162 (50.5)	1.00
Heart failure reduced ejection fraction	103 (67.3)	1,077,899 (24.0)	<0.0001	71 (66.4)	213 (66.4)	1.00

ACEI = angiotensin-converting enzyme inhibitors.

By matching patient demographics and risk indicators, we were able to create an analytic cohort consisting of 107 IVB patients and 321 matched controls. The matching process eliminates the patients with extremely high risk from the IVB groups while selecting the patients with elevated risk profiles from the control groups. The mean (SD) year age for IVB and matched controls after matching was 52.9 (11.3) and 53.5 (11.8), respectively, with the proportion of middle-aged patients of 76.6% for both IVB and control groups. The proportion of HF-related medications and cardiovascular risk indicators was exactly matched (Table 1).

The Kaplan-Meier estimates from the matched cohort indicated that the cumulative incidence of AF at 180 days after the index date for IVB and controls were 55.4% and 10.1%, respectively. The HR estimate (95% CI) from the Cox proportional hazard regression model was 7.293 (4.985-10.668) (Central Illustration).

**Central Illustration:**
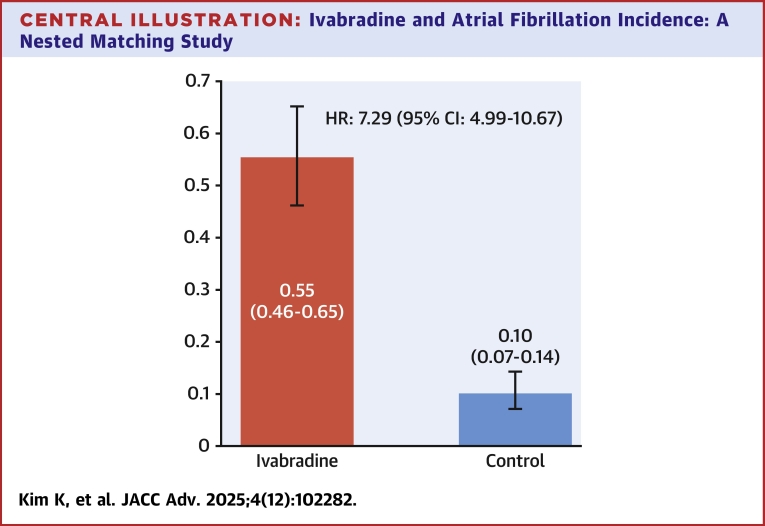
**Ivabradine and Atrial Fibrillation Incidence: A Nested Matching Study** Cumulative incidence of atrial fibrillation at 180 days after the use of ivabradine or matched control date and HR estimate.

## Discussion

In a nested-matching study from a U.S.-based commercial insurance and Medicare supplement claims database, HF patients exposed to IVB had an increase in the cumulative incidence of AF when compared to the patients who had not received IVB. This association remained significant even after adjusting for confounding factors captured in the health care claims. On the backdrop of extensive use of beta-blockers and blockade of angiotensin in our study population and control group, AF incidence rates compared to those reported in patients with NYHA functional classification II-III (IVB indicated use),^12^ these results suggest that the incidence of AF in HF patients based on real-world data may be a more common than previously reported in clinical trials.^13^, ^14^, ^15^, ^16^, ^17^

**Figure 2:**
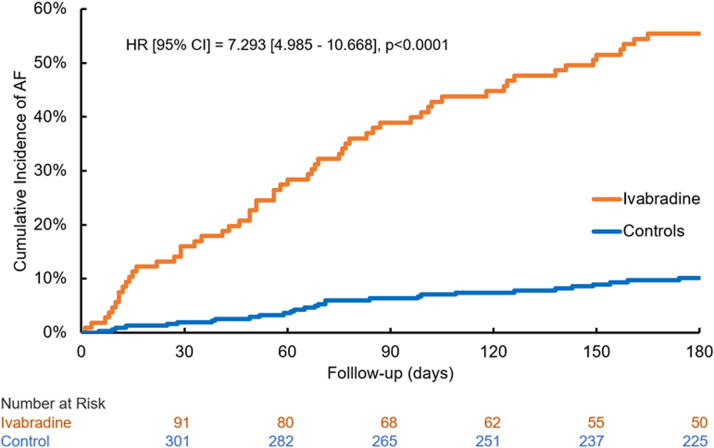
**Cumulative Incidence of AF and HR Estimate** Cumulative incidence of AF calculated from Kaplan-Meier estimate; HR of the onset of AF for ivabradine vs control was calculated from a multivariable Cox proportional hazard regression model. Orange line, cumulative incidence of AF in patients receiving ivabradine; blue line, cumulative incidence of AF in matched controls who were not on ivabradine. Abbreviation as in Figure 1.

IVB-associated AF incidence may be an association rather than a cause-effect relationship. However, regulation of heart rate by IVB occurs through blockade of the cardiac pacemaker current (HCN funny current) [I_f_].^18^ Pulmonary vein myocardial sleeves which are notable AF triggers have I_f_ currents, which may impact AF incidence.^19^ IVB promotes arrhythmias immediately after initiation of the medication (Figure 2) suggesting that the effect is not dose-related. Hence, the combination of IVB pharmacological effect coupled with either genetic variability and/or intrinsic physiological heterogeneities in tissue composition may contribute to the proarrhythmic effect of the agent.^20^

In previous clinical trials of IVB for U.S. Food and Drug Administration approval,^15^^,^^16^ AF was an exclusion criterion. In a multicenter, observational matched cohort study of patients with symptomatic HFrEF and a history of paroxysmal AF, 887 patients prescribed IVB were compared to 1115 with no IVB. In the study, IVB treatment was associated with an increased risk of the primary outcome of HF hospitalization and HF death (HR: 1.56; 95% CI: 1.40-1.75 and HR: 1.67; 95% CI: 1.14-re2.44).^21^ These results, combined with our findings, suggest that further trials are needed to understand the risks of IVB in HF patients.

### Confounding by indication

Any interpretation should carefully consider confounding by indication—the major concern in observational research with passive controls from retrospective identification. While our cohort selection strategies decreased differences between groups, unreported patient characteristics, measures of HF severity, and other unmeasured factors likely predispose patients to both IVB prescription and AF, raising concerns about confounding by indication. Therefore, the HR estimates may not accurately reflect the risk of AF directly associated with exposure to IVB. Various methods should be utilized when confounding by indication is a major concern, such as the instrumental variable approach or negative control outcomes.^22^^,^^23^ However, due to the lack of such information from the claims database, further attempts to minimize concerns about confounding by indication could not be operationalized.

Nevertheless, the findings provide noteworthy information. The matched patient characteristics and incidence density sampling approach alleviated concerns about observable confounders and the influence of HF progression attributable to the duration of living with HF. Particularly, by matching the previous history of hospital admissions and diagnosis of HFrEF, the factors that could have been captured at the administrative level have already been addressed in our study. In addition, we found the cumulative incidence exceeded 50% among IVB patients, and the HR was >7. The anticipated impact of confounding would not be large enough to negate our entire measure of association. Our study suggests that the risk of AF with IVB should be considered when planning patient surveillance and warrants additional study.

### Study limitations

There are several limitations to this study. Firstly, the accuracy of identifying patients and outcomes is subject to the performance of algorithms involving the diagnosis code use.^24^ Using diagnosis codes for AF attained the positive predictive value ranged from 55% to 90% depending on the diagnosis position and setting, which raises questions on the accuracy of the estimated incidence.^25^ While applying more specific algorithm, such as 2 or more diagnosis in 2 different dates, might improve the proportion of true-positive cases, replacing our single diagnosis use with multiencounter algorithms can significantly decrease the event count particularly when left censoring could happen before patient encountered with a follow-up visit. Furthermore, the influence of this misclassification would be nondifferential, which means that the anticipated impact of the algorithm accuracy on the magnitude and direction of the association, as measured by the HR, is expected to be nominal. Therefore, even in the worst-case scenario, the anticipated impact of the algorithm accuracy would be nominal in the magnitude and direction of the association calculating HR. Another limitation would be not including mortality, which could serve as a competing event prior to the onset of AF. We acknowledge that such limitation could not be fully addressed due to the lack of our capacity in collecting death records from the existing database, while the mortality impact would not be large enough to decrease the magnitude of the association. Also, the dose-event association could not be fully addressed from our analysis, since the discrepancy between the dispensing records and prescribed regimen and actual persistence. Other general limitations including miscoding, missing records, unobserved confounders, and limited generalizability should also be considered. The issue of confounding by indication has been addressed in detail.

## Conclusions

In HF AF-naive patients, analysis of health care claims suggest a significant risk of developing AF among patients who received IVB. However, further studies are warranted to understand this association, including the impact of unmeasured risk factors and confounding by indication.

Perspectives **COMPETENCY IN MEDICAL KNOWLEDGE:** In HF AF-naive patients, this study using health care claims suggests a significant risk of developing AF when patients are prescribed IVB. These findings *highlight* the need for further studies to understand the risk of AF in HF patients treated with IVB.

## Funding support and author disclosures

This work was supported by 10.13039/100007747University of Utah Cardiovascular Clinical Pharmacology Research Fund. The authors have reported that they have no relationships relevant to the contents of this paper to disclose.
